# A multi-centre longitudinal study analysing multiple sclerosis disease-modifying therapy prescribing patterns during the COVID-19 pandemic

**DOI:** 10.1007/s00415-024-12518-7

**Published:** 2024-06-27

**Authors:** Anoushka P. Lal, Yi Chao Foong, Paul G. Sanfilippo, Tim Spelman, Louise Rath, David Levitz, Marzena Fabis-Pedrini, Matteo Foschi, Mario Habek, Tomas Kalincik, Izanne Roos, Jeannette Lechner-Scott, Nevin John, Aysun Soysal, Emanuele D’Amico, Riadh Gouider, Saloua Mrabet, Katrin Gross-Paju, Simón Cárdenas-Robledo, Abdorreza Naser Moghadasi, Maria Jose Sa, Orla Gray, Jiwon Oh, Stephen Reddel, Sudarshini Ramanathan, Talal Al-Harbi, Ayse Altintas, Todd A. Hardy, Serkan Ozakbas, Raed Alroughani, Allan G. Kermode, Andrea Surcinelli, Guy Laureys, Sara Eichau, Alexandre Prat, Marc Girard, Pierre Duquette, Suzanne Hodgkinson, Cristina Ramo-Tello, Davide Maimone, Pamela McCombe, Daniele Spitaleri, Jose Luis Sanchez-Menoyo, Mehmet Fatih Yetkin, Seyed Mohammad Baghbanian, Rana Karabudak, Abdullah Al-Asmi, Gregor Brecl Jakob, Samia J. Khoury, Masoud Etemadifar, Vincent van Pesch, Katherine Buzzard, Bruce Taylor, Helmut Butzkueven, Anneke Van der Walt

**Affiliations:** 1 Department of Neuroscience, Central Clinical School, The Alfred, Melbourne, VIC Australia; 2 Department of Neurology, The Alfred Hospital, 55 Commercial Road, Melbourne, 3004 Australia; 3 Perron Institute for Neurological and Translational Science, The University of Western Australia, Perth, Australia; 4 Centre for Molecular Medicine and Innovative Therapeutics, Murdoch University, Perth, Australia; 5 Department of Neuroscience, MS Center, Neurology Unit, S. Maria Delle Croci Hospital, AUSL Romagna, Ravenna, Italy; 6 Department of Biotechnological and Applied Clinical Sciences (DISCAB), University of L’Aquila, L’Aquila, Italy; 7 Department of Neurology, University Hospital Center Zagreb, Zagreb, Croatia; 8 School of Medicine, University of Zagreb, Zagreb, Croatia; 9 Department of Neurology, Neuroimmunology Centre, Royal Melbourne Hospital, Melbourne, Australia; 10 CORe, Department of Medicine, University of Melbourne, Melbourne, Australia; 11 Hunter Medical Research Institute, University Newcastle, Newcastle, Australia; 12 Department of Medicine, School of Clinical Sciences, Monash University, Clayton, Australia; 13 Department of Neurology, Monash Health, Clayton, Australia; 14 Bakirkoy Education and Research Hospital for Psychiatric and Neurological Diseases, Istanbul, Turkey; 15 Medical and Surgical Sciences, Universita Di Foggia, Foggia, Italy; 16 Department of Neurology, LR 18SP03, Clinical Investigation Centre Neurosciences and Mental Health, Razi University Hospital, Tunis, Tunisia; 17 Faculty of Medicine of Tunis, University of Tunis El Manar, Tunis, Tunisia; 18 Multiple Sclerosis Centre, West-Tallinn Central Hospital, Tallinn, Estonia; 19 Department of Neurology, Centro de Esclerosis Múltiple (CEMHUN), Hospital Universitario Nacional de Colombia Bogota, Bogota, Colombia; 20 Departamento de Medicina InternaFacultad de Medicina, Universidad Nacional de Colombia, Bogota, Colombia; 21 Multiple Research Centre, Neuroscience Institute, Tehran University of Medical Science, Tehran, Iran; 22 Department of Neurology, Centro Hospitalar Universitario de Sao Joao, Porto, Portugal; 23 South Eastern HSC Trust, Belfast, UK; 24 St. Michael’s Hospital, Toronto, Canada; 25 Department of Neurology, Concord Repatriation General Hospital, Sydney, Australia; 26 Translational Neuroimmunology Group, Kids Neuroscience Centre and Brain and Mind Centre, Concord Hospital, Sydney, Australia; 27 Neurology Department, King Fahad Specialist Hospital-Dammam, Dammam, Saudi Arabia; 28 Department of Neurology, School of Medicine and Koc University Research Center for Translational Medicine (KUTTAM), İstanbul, Turkey; 29 Izmir University of Economics, Medical Point Hospital, Izmir, Turkey; 30 Multiple Sclerosis Research Association, Izmir, Turkey; 31 Division of Neurology, Department of Medicine, Amiri Hospital, Sharq, Kuwait; 32 Department of Neurology, University Hospital Ghent, Ghent, Belgium; 33 Department of Neurology, Hospital Universitario Virgen Macarena, Seville, Spain; 34 CHUM and Universite de Montreal, Montreal, Canada; 35 Immune Tolerance Laboratory Ingham Institute and Department of Medicine, UNSW, Sydney, Australia; 36 Department of Neuroscience, Hospital Germans Trias I Pujol, Badalona, Spain; 37 Centro Sclerosi Multipla, UOC Neurologia, Azienda Ospedaliera Per L’Emergenza Cannizzaro, Catania, Italy; 38 University of Queensland, Brisbane, Australia; 39 Royal Brisbane and Women’s Hospital, Brisbane, Australia; 40 Azienda Ospedaliera Di Rilievo Nazionale San Giuseppe Moscati Avellino, Avellino, Italy; 41 Department of Neurology, Galdakao-Usansolo University Hospital, Osakidetza-Basque Health Service, Galdakao, Spain; 42 Department of Neurology, Erciyes University, Kayseri, Turkey; 43 Neurology Department, Booalisina Hospital, Mazandaran University of Medical Sciences, Sari, Iran; 44 Faculty of Medicine, Mazandaran University of Medical Sciences, Sari, Iran; 45 Department of Neurological Sciences, Faculty of Medicine, Yeditepe University, Istanbul, Turkey; 46 Neuroimmunology Unit, Koşuyolu Hospitals, Istanbul, Turkey; 47 College of Medicine & Health Sciences and Sultan Qaboos University Hospital, Sultan Qaboos University, Al-Khodh, Oman; 48 Department of Neurology, University Medical Centre Ljubljana, Ljubljana, Slovenia; 49 Faculty of Medicine, University of Ljubljana, Ljubljana, Slovenia; 50 Nehme and Therese Tohme Multiple Sclerosis Center, American University of Beirut Medical Center, Beirut, Lebanon; 51 Neurology, Dr. Etemadifar MS Institute, Isfahan University of Medical Sciences, Isfahan, Iran; 52 Department of Neurology, Cliniques Universitaires Saint-Luc, Brussels, Belgium; 53 Department of Neurosciences, Box Hill Hospital, Box Hill, Australia; 54 Royal Hobart Hospital, Hobart, Australia

**Keywords:** Multiple sclerosis, COVID-19, Disease-modifying therapy, Anti-CD20 monoclonal antibodies, Cladribine, Natalizumab

## Abstract

**Background:**

The COVID-19 pandemic raised concern amongst clinicians that disease-modifying therapies (DMT), particularly anti-CD20 monoclonal antibodies (mAb) and fingolimod, could worsen COVID-19 in people with multiple sclerosis (pwMS). This study aimed to examine DMT prescribing trends pre- and post-pandemic onset.

**Methods:**

A multi-centre longitudinal study with 8,771 participants from MSBase was conducted. Two time periods were defined: pre-pandemic (March 11 2018–March 10 2020) and post-pandemic onset (March 11 2020–11 March 2022). The association between time and prescribing trends was analysed using multivariable mixed-effects logistic regression. DMT initiation refers to first initiation of any DMT, whilst DMT switches indicate changing regimen within 6 months of last use.

**Results:**

Post-pandemic onset, there was a significant increase in DMT initiation/switching to natalizumab and cladribine [(Natalizumab-initiation: OR 1.72, 95% CI 1.39–2.13; switching: OR 1.66, 95% CI 1.40–1.98), (Cladribine-initiation: OR 1.43, 95% CI 1.09–1.87; switching: OR 1.67, 95% CI 1.41–1.98)]. Anti-CD20mAb initiation/switching decreased in the year of the pandemic, but recovered in the second year, such that overall odds increased slightly post-pandemic (initiation: OR 1.26, 95% CI 1.06–1.49; Switching: OR 1.15, 95% CI 1.02–1.29. Initiation/switching of fingolimod, interferon-beta, and alemtuzumab significantly decreased [(Fingolimod-initiation: OR 0.55, 95% CI 0.41–0.73; switching: OR 0.49, 95% CI 0.41–0.58), (Interferon-gamma-initiation: OR 0.48, 95% CI 0.41–0.57; switching: OR 0.78, 95% CI 0.62–0.99), (Alemtuzumab-initiation: OR 0.27, 95% CI 0.15–0.48; switching: OR 0.27, 95% CI 0.17–0.44)].

**Conclusions:**

Post-pandemic onset, clinicians preferentially prescribed natalizumab and cladribine over anti-CD20 mAbs and fingolimod, likely to preserve efficacy but reduce perceived immunosuppressive risks. This could have implications for disease progression in pwMS. Our findings highlight the significance of equitable DMT access globally, and the importance of evidence-based decision-making in global health challenges.

## Background

The COVID-19 pandemic caused a multitude of unprecedented challenges in healthcare systems across the globe. Amongst the vulnerable populations affected by the COVID-19 pandemic were people with multiple sclerosis (pwMS). The overall COVID-19 mortality rate amongst patients with either suspected or confirmed MS was estimated to be around 3.0% [1].

In general, pwMS, especially those on disease-modifying therapies (DMT), are more susceptible to infectious diseases and are at a higher risk of infection-related hospitalisations compared to the general population [2]. Specifically for COVID-19, older age, African American ethnicity, and a higher level of disability all significantly increase the risk of experiencing severe infections amongst pwMS [1, 3, 4]. A crucial additional risk factor identified for severe COVID-19 infections in pwMS was the use of certain immunosuppressive DMTs. This posed a significant challenge in MS care for clinicians and led to various consensus agreements and recommendations being published [5–7]. General consensus suggested that lower efficacy DMTs such as interferons and glatiramer acetate were unlikely to increase the risk of severe COVID-19 infection and, potentially, that interferon DMTs may even be protective [8]. However, higher efficacy medications, particularly anti-CD20 monoclonal antibodies (such as ocrelizumab and rituximab) and S1P inhibitors (such as fingolimod), were considered to potentially increase the susceptibility to as well as the severity of COVID-19 for pwMS [9–12].

Current literature suggests that there was a shift in DMT prescribing patterns in pwMS during the COVID-19 pandemic. There was a significant reduction in the initiation of high-efficacy immunosuppressive DMTs, such as anti-CD20 monoclonal antibodies and S1P inhibitors [13–15]. Instead, there was an increased preference for lower efficacy, self-injectable DMTs such as interferon-beta and glatiramer acetate, which were perceived as safer options during the pandemic [13, 16]. Despite the overall reduction in high-efficacy DMT prescriptions, some clinicians continued or initiated these therapies with modifications, such as extended interval dosing, to reduce the risk of severe infections whilst maintaining disease control [17, 18].

These studies, however, were limited by sample size and country-based variation in practice, and the implications of these changes on disease activity in pwMS are yet to be fully elucidated [15]. In this study, we performed a longitudinal multi-centre study across over 25 countries using the MSBase Registry to evaluate prescription patterns of DMTs and to analyse the impact of the COVID-19 pandemic on the care of pwMS.

## Methods

### Participant selection and patient consent

We conducted a multi-centre, retrospective study using 8,771 participants from the MSBase Registry. All participants provided written informed consent to be a part of the study. Ethics approval for the MSBase registry was granted by the Alfred Health Human Research and Ethics Committee and the local ethics committees of all the participating centres that comprise the MSBase. This study followed the Strengthening the Reporting of Observational Studies in Epidemiology (STROBE) reporting guidelines.

### Study participants

The inclusion criteria for this study were: (1) 18 years of age or over; (2) definite diagnosis of MS according to the McDonald Criteria [21]; and (3) at least one visit recorded in the pre-pandemic or post-pandemic period AND at least one visit after 11 March 2022. Patients with incomplete demographic (sex, age) or clinic (disease duration, the date of starting and/or stopping DMTs, Expanded Disability Status Scale, EDSS, assessments and dates of relapses for the duration of the study) were excluded.

Two time periods were defined as: (1) pre-pandemic (March 11 2018 to March 10 2020) and (2) post-pandemic (March 11 2020, when the COVID-19 pandemic was announced by World Health Organisation, to 11 March 2022) [18].

### Outcomes and definitions

The primary outcome was to analyse the prescribing patterns of high- and low-efficacy DMTs pre- and post-pandemic onset. We classified initiation and switching to DMT as follows: DMT initiation referred to the first prescription of any DMT. DMT switching referred to change in DMT regimen within 6 months of last DMT use.

High-medium efficacy DMTs (called high-efficacy from here on) were defined as ocrelizumab, rituximab, ofatumumab, cladribine, alemtuzumab, natalizumab, and fingolimod. Low-efficacy DMTs were defined as interferon-beta/alpha, glatiramer acetate, teriflunomide and dimethyl fumarate (DMF). To reduce groups for comparison, interferon-beta/alpha and glatiramer acetate were grouped together as “BRACE”, and rituximab, ocrelizumab and ofatumumab were grouped as “Anti-CD20 mAbs”.

### Statistical analysis

The demographic information and the baseline characteristics were reported as number and percentage for discrete variables and as mean (standard deviation [SD]) or median (interquartile range [IQR]) for continuous variables, as appropriate and according to the data distribution.

Using a pre-post design, we applied generalised linear mixed models with a binomial link function and a random effect for each country to assess associations between DMT initiation or switching (outcomes) as a function of DMT class across pandemic periods (exposures). In the models, the random effect was country of residence, whilst fixed covariates were age, gender, MS phenotype, disease duration, EDSS, and relapse count in the previous 24 months. All statistical tests were two-sided with a statistical significance defined as *p* ≤ 0.05. Analyses were performed in R version.4.3.0. (R Foundation for Statistical Computing).

## Results

### Participant demographics

8,771 participants were selected for this study. There were 4,533 initiations and 5899 switches recorded in 5165 unique individuals. Note that this discrepancy in sample numbers arises from instances where some participants may have had an initiation of DMT followed by a subsequent switch, thus contributing to both counts. Table 1 outlines the participant demographics for total participants and participants where initiations and switches were recorded.

**Table 1:** Participant demographics

Characteristic	Total *n* = 8771 (%)	Initiation *n* = 4533 (%)	Switching *n* = 5165 (%)
Gender			
F	6,222 (71%)	3,150 (69%)	3,747 (73%)
M	2,549 (29%)	1,383 (31%)	1,418 (27%)
Age category (years)			
0–20	326 (3.7%)	257 (5.7%)	147 (2.8%)
21–30	1,682 (19%)	1,130 (25%)	841 (16%)
31–40	2,556 (29%)	1,386 (31%)	1,455 (28%)
41–50	2,318 (26%)	1,011 (22%)	1,490 (29%)
51–60	1,392 (16%)	520 (11%)	944 (18%)
> 60	497 (5.7%)	229 (5.1%)	288 (5.6%)
Country			
Australia	2,512 (29%)	1,293 (29%)	1,414 (27%)
Turkey	1,991 (23%)	927 (20%)	1,346 (26%)
Italy	706 (8.0%)	442 (9.8%)	352 (6.8%)
Spain	590 (6.7%)	288 (6.4%)	364 (7.0%)
Kuwait	559 (6.4%)	353 (7.8%)	244 (4.7%)
Iran	429 (4.9%)	163 (3.6%)	294 (5.7%)
Croatia	309 (3.5%)	234 (5.2%)	121 (2.3%)
Belgium	267 (3.0%)	150 (3.3%)	161 (3.1%)
Tunisia	103 (1.2%)	74 (1.6%)	47 (0.9%)
Japan	94 (1.1%)	70 (1.5%)	43 (0.8%)
Netherlands	87 (1.0%)	37 (0.8%)	59 (1.1%)
Other*	497 (5.7%)	274 (6.0%)	273 (5.3%)
MS course		1,293 (29%)	
Relapsing remitting	7,610 (87%)	3,906 (86%)	4,570 (88%)
Secondary progressive	537 (6.1%)	113 (2.5%)	443 (8.6%)
Primary progressive	303 (3.5%)	253 (5.6%)	66 (1.3%)
Progressive relapsing	95 (1.1%)	60 (1.3%)	42 (0.8%)
Radiologically isolated syndrome	4 (< 0.1%)	3 (< 0.1%)	2 (< 0.1%)
Clinically isolated syndrome	222 (2.5%)	198 (4.4%)	42 (0.8%)
EDSS	2.4 (1.9%)	2.0 (1.7%)	2.6 (2.0%)
Relapse count (in previous 24 months)			
0	4,708 (54%)	2,016 (44%)	3,005 (58%)
1	2,847 (32%)	1,790 (39%)	1,478 (29%)
2	918 (10%)	570 (13%)	498 (9.6%)
3	228 (2.6%)	126 (2.8%)	137 (2.7%)
4	46 (0.5%)	23 (0.5%)	28 (0.5%)
5	16 (0.2%)	7 (0.2%)	12 (0.2%)
6	3 (< 0.1%)	1 (< 0.1%)	2 (< 0.1%)
7	4 (< 0.1%)	-	4 (< 0.1%)
10	1 (< 0.1%)	-	1 (< 0.1%)
Disease duration (years)	8.2 (8.3%)	4.4 (6.7%)	10.6 (8.3%)

^*^See appendix for full list of countries

### Comparison of high- and low-efficacy DMT prescription pre- and post-pandemic onset

There was an overall decrease in initiating and switching DMTs post-pandemic compared to pre-pandemic (Table 2). There was a significant increase in initiation of low-efficacy DMTs post-pandemic compared to pre-pandemic (54.1 to 59.6%) and a decrease in initiation of high-efficacy DMTs (45.9 to 40.4%). There was a significant increase in switching to low-efficacy DMTs post-pandemic (27.4 to 29%) and a decrease in switching to high-efficacy DMTs post-pandemic (72.6 to 71%).

**Table 2:** Comparison of initiation and switches pre- and post-pandemic with low-efficacy and high-efficacy DMTs

	Initiation			Switching		
	Overall *N* = 4,533	Pre-pandemic *N* = 2,443	Post-pandemic *N* = 2,090	Overall *N* = 5,899	Pre-pandemic *N* = 3,306	Post-pandemic *N* = 2,593
Low-efficacy DMT	2,568 (56.6%)	1,322 (54.1%)	1,246 (59.6%)	1,657 (28.1%)	906 (27.4%)	751 (29%)
High-efficacy DMT	1,965 (43.4%)	1,121 (45.9%)	844 (40.4%)	4,242 (71.9%)	2,400 (72.6%)	1,842 (71.0%)

### Analysis of DMT prescribing patterns pre- and post-pandemic onset

**Fig. 1:**
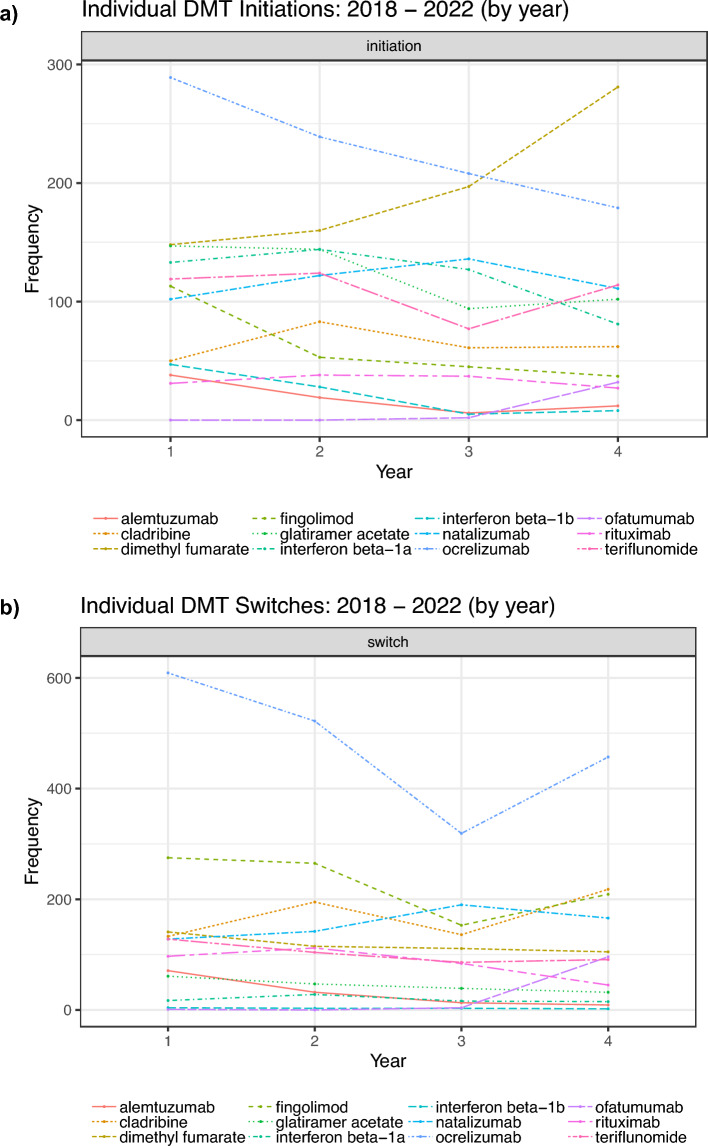
Patterns of frequency of DMT initiation (**a**) and DMT switching (**b**) divided by year. Year 1 represents March 2018 to March 2019, Year 2 represents March 2019 to March 2020, Year 3 represents March 2021 to March 2022, and Year 4 represents March 2021 to March 2022

Post-pandemic onset, there was an increase in DMT initiation and switching to natalizumab (OR 1.72, 95% CI 1.39–2.13; OR 1.66, 95% CI 1.40–1.98) and cladribine (OR 1.43, 95% CI 1.09–1.87; OR 1.67, 95% CI 1.41–1.98) (Table 3). The initiation and switching to anti-CD20 monoclonal antibodies (mABs) decreased immediately following the onset of the COVID-19 pandemic in 2020; however, there was a steady increase towards the end of the pandemic, resulting in an overall rise in initiation and switching to anti-CD20 mABs (OR 1.26, 95% CI 1.06–1.49; OR 1.15, 95% CI 1.02–1.29) (Fig. 1). This increase was statistically significant but relatively smaller increase compared to natalizumab or cladribine.

**Table 3:** Comparison of DMT initiation and switching from pre- to post-pandemic

	Initiation—OR (95% CI)	Switching—OR (95% CI)
Anti-CD20 mAb		
Ocrelizumab, ofatumumab and rituximab	1.26 (1.06–1.49)	1.15 (1.02–1.29)
Oral immunomodulators		
Dimethyl fumarate	1.76 (1.49–2.09)	0.85 (0.69–1.05)
Teriflunomide	0.77 (0.62–0.96)	0.80 (0.64–0.99)
Fingolimod	0.55 (0.41–0.73)	0.49 (0.41–0.58)
Injectable immunomodulators		
BRACE	0.78 (0.62–0.99)	0.78 (0.62–0.99)
Integrin antagonist		
Natalizumab	1.72 (1.39–2.13)	1.66 (1.40–1.98)
Purine analogue		
Cladribine	1.43 (1.09–1.87)	1.67 (1.41–1.98)
Other		
Alemtuzumab	0.27 (0.15–0.48)	0.27 (0.17–0.44)

BRACE includes interferon-beta and glatiramer acetate, and anti-CD20 mAbs includes rituximab, ocrelizumab and ofatumumab. Data are displayed with Odds ratios and 95% CI

There was a decrease in initiating and switching patients to fingolimod (OR 0.55, 95% CI 0.41–0.73; OR 0.49, 95% CI 0.41–0.58), interferon-beta (OR 0.48, 95% CI 0.41–0.57; OR 0.78, 95% CI 0.62–0.99) and alemtuzumab (OR 0.27, 95% CI 0.15–0.48; OR 0.27, 95% CI 0.17–0.44) post-pandemic onset (Table 3). There was an increase in initiating (OR 1.76, 95% CI 1.49–2.09), but a decrease in switching (OR 0.85, 95% CI 0.69–1.05) patients to DMF.

## Discussion

We performed a retrospective multi-site analysis of the prescribing patterns of DMTs in pwMS after the onset of the COVID-19 pandemic. We hypothesised there would be a significant decrease in DMT prescription, particularly anti-CD20 mAbs and fingolimod, given concerns regarding immunosuppression during the COVID-19 pandemic. Our results demonstrate a significant decrease in initiation and switching to fingolimod, alemtuzumab and interferon-beta post-pandemic onset, and a significant increase in initiation and switching patients to natalizumab and cladribine There was also a slight increase to anti-CD20 mAbs (though notably less than other higher-efficacy DMTs). This supports our hypothesis that concerns around more severe COVID-19 outcomes for pwMS on fingolimod and anti-CD20 mAbs influenced prescribing patterns during the pandemic. The increased usage of natalizumab and cladribine post-pandemic onset was likely driven by clinicians attempting to maintain prescribing high-efficacy treatments for patients but avoiding the usage of anti-CD20 mAb therapies due to perceived immunosuppression risks.

Our results show an overall decrease in the initiation and switching of DMTs during the COVID-19 pandemic. We specifically observed an approximate 5% decrease in initiation and 1% decrease in switching patients to high-efficacy DMTs post-pandemic onset, which was likely driven by clinicians choosing to reduce the prescribing of high-efficacy treatments based on concerns of worsening COVID-19 susceptibility and severity in patients. This is consistent with retrospective cohort studies, which have noted an overall decrease in DMT prescription or a change in dosing regimen, specifically in high-efficacy DMTs [13, 16]. This significant shift in underutilisation of higher efficacy DMTs and increased initiation and switching to lower efficacy DMTs has significant implications for relapse probability in pwMS at a population scale and could have a negative impact on overall health outcomes for pwMS [22].

Our results reveal that clinicians increased the prescription of cladribine and natalizumab during the COVID-19 pandemic, likely as they were considered safer, high-efficacy treatments for pwMS. Current evidence indicates that cladribine does not increase susceptibility to COVID-19 infections or exacerbate infection severity. Case studies have shown that pwMS treated with cladribine mount an appropriate immunological response and typically experience mild symptoms following COVID-19 infection [23–25]. This may be due to the immune reconstitution properties of cladribine, wherein it causes selectively transient reductions in CD19^+^ B and T cells, followed by reconstitution and restoration of the body’s adaptive immunity [26]. Natalizumab is not associated with worse COVID-19 clinical outcomes [27, 28]. Indeed, there is some postulation that natalizumab may be protective against COVID-19 infection by limiting viral entry into cells through the integrin blockade [29, 30].

Anti-CD20 mAb initiation and switching decreased in 2020. Still, it returned to pre-pandemic levels in 2021, such that overall, there was a slight increase in anti-CD20 mAb prescription (though less than other high-efficacy DMTs). These results are consistent with other studies that also observed a decrease in prescribing anti-CD20 mAbs during the COVID-19 pandemic [14]. Multiple cohort studies have indicated a significant relationship between anti-CD20 mAbs and increased severity of COVID-19 infection, thereby indicating the rapid response from clinicians to avoid prescribing, initiating or switching patients to these therapies was appropriate from a COVID-19 disease severity perspective [17, 18, 31, 32].

Concerns with its lymphopenic effects may have driven the decrease in initiating and switching to fingolimod as it is specifically associated with higher rates of Herpes Zoster virus infections compared to other DMTs [33, 34]. Whilst individual case studies have suggested a potential increase in the severity of COVID-19 infections with fingolimod use, larger cohort studies and current evidence indicate that fingolimod is not associated with worse COVID-19 outcomes or increased hospitalisation rates [32, 35–37]. The continued decrease in usage of fingolimod in 2021 implies that clinicians might be progressively opting for other high-efficacy DMTs over fingolimod.

The decrease in initiation and switching patients to alemtuzumab was likely due to published recommendations at the start of the pandemic, which suggested delaying lymphodepleting treatments such as alemtuzumab until the COVID-19 pandemic was more controlled [7]. In addition, prescription may have been influenced by limited access to inpatient healthcare services as various countries navigated the pandemic with various lockdown restrictions.

The increase in DMF initiation may have been based on recommendations from early guidelines that were published at the start of the pandemic, which encouraged the prescription of first-line DMTs such as teriflunomide and dimethyl fumarate [7]. The observed decrease in initiation and switching to interferon treatments post-pandemic onset may have been driven by concerns over immune system modulation and a preference for oral medications during the pandemic due to decreased access to healthcare facilities.

The limitations of our study include the fact that we did not record the reasons for clinicians’ choices of DMT initiation or switching. Patients’ co-morbidities and COVID-19 vaccine status were not recorded, which could have influenced DMT choice. Furthermore, concerns regarding DMTs affecting vaccine efficacy may also have influenced DMT therapy initiation choices [38, 39]. The temporal dynamics of the pandemic which varied substantially between countries, characterised by various waves and changing public health responses, alongside the evolution in the availability and types of COVID-19 vaccines, may have also influenced clinicians’ choices for DMT initiation and switching. In addiiton, individual access to DMTs, which varied greatly between countries and supply chain issues, may have influenced prescribing preferences.

Considered collectively, it is evident that during the COVID-19 pandemic, there was a nuanced balance between mitigating severe COVID-19 infections and ensuring continued use of high-efficacy DMTs to minimise MS disease activity. The approach to prescribing DMTs for pwMS demonstrated a significant evolution from initial, recommendation-driven practices to more robust, data-driven strategies. The initial hesitancy to prescribe certain high-efficacy DMTs, such as anti-CD20 monoclonal antibodies and fingolimod, was influenced by concerns regarding their impact on COVID-19 severity and vaccine efficacy. Over time, however, prescribing patterns adapted in response to accumulating clinical evidence, highlighting the resilience and adaptability of clinicians in managing treatment of pwMS under global health challenges.

Moreover, this shift highlights a crucial need for international equity in access to DMTs. Our findings suggest that the ability to select the most appropriate therapy based on up-to-date evidence was at times limited by availability and accessibility, affecting treatment choices globally. As such, ensuring equitable access to a range of DMTs is essential, not only for managing MS more effectively but also for preparing healthcare systems to respond more effectively to future global health emergencies. Our research highlights the necessity of evidence-based decision-making and collaborative efforts amongst researchers, clinicians, and healthcare systems to optimise care and protect the health outcomes of pwMS amid ongoing global health challenges.

## Appendix 1

See Table

**Table 4:** Participant demographics

Characteristic	Total *n* = 8771 (%)	Initiation *n* = 4533 (%)	Switching *n* = 5165 (%)
Gender			
F	6,222 (71%)	3,150 (69%)	3,747 (73%)
M	2,549 (29%)	1,383 (31%)	1,418 (27%)
Age category (years)			
0–20	326 (3.7%)	257 (5.7%)	147 (2.8%)
21–30	1,682 (19%)	1,130 (25%)	841 (16%)
31–40	2,556 (29%)	1,386 (31%)	1,455 (28%)
41–50	2,318 (26%)	1,011 (22%)	1,490 (29%)
51–60	1,392 (16%)	520 (11%)	944 (18%)
> 60	497 (5.7%)	229 (5.1%)	288 (5.6%)
Country			
AU	2,512 (29%)	1,293 (29%)	1,414 (27%)
AE	30 (0.3%)	15 (0.3%)	18 (0.3%)
BE	267 (3.0%)	150 (3.3%)	161 (3.1%)
CA	627 (7.1%)	228 (5.0%)	447 (8.7%)
CO	68 (0.8%)	63 (1.4%)	16 (0.3%)
EE	70 (0.8%)	34 (0.8%)	45 (0.9%)
ES	590 (6.7%)	288 (6.4%)	364 (7.0%)
GB	38 (0.4%)	13 (0.3%)	29 (0.6%)
GR	4 (< 0.1%)	1 (< 0.1%)	3 (< 0.1%)
HR	309 (3.5%)	234 (5.2%)	121 (2.3%)
HU	2 (< 0.1%)	2 (< 0.1%)	1 (< 0.1%)
IR	429 (4.9%)	163 (3.6%)	294 (5.7%)
IT	706 (8.0%)	442 (9.8%)	352 (6.8%)
JP	94 (1.1%)	70 (1.5%)	43 (0.8%)
KW	559 (6.4%)	353 (7.8%)	244 (4.7%)
LB	45 (0.5%)	20 (0.4%)	31 (0.6%)
NL	87 (1.0%)	37 (0.8%)	59 (1.1%)
NZ	28 (0.3%)	17 (0.4%)	14 (0.3%)
OM	53 (0.6%)	23 (0.5%)	37 (0.7%)
Other	107 (1.2%)	72 (1.6%)	40 (0.8%)
PL	1 (< 0.1%)	1 (< 0.1%)	0 (0%)
PT	50 (0.6%)	12 (0.3%)	39 (0.8%)
TN	103 (1.2%)	74 (1.6%)	47 (0.9%)
TR	1,991 (23%)	927 (20%)	1,346 (26%)
US	1 (< 0.1%)	1 (< 0.1%)	0 (0%)
MS course		1,293 (29%)	
Relapsing remitting	7,610 (87%)	3,906 (86%)	4,570 (88%)
Secondary progressive	537 (6.1%)	113 (2.5%)	443 (8.6%)
Primary progressive	303 (3.5%)	253 (5.6%)	66 (1.3%)
Progressive relapsing	95 (1.1%)	60 (1.3%)	42 (0.8%)
Radiologically isolated syndrome	4 (< 0.1%)	3 (< 0.1%)	2 (< 0.1%)
Clinically isolated syndrome	222 (2.5%)	198 (4.4%)	42 (0.8%)
EDSS	2.37 (1.88)	1.99 (1.67)	2.64 (1.96)
Relapse count (in previous 24 months)			
0	4,708 (54%)	2,016 (44%)	3,005 (58%)
1	2,847 (32%)	1,790 (39%)	1,478 (29%)
2	918 (10%)	570 (13%)	498 (9.6%)
3	228 (2.6%)	126 (2.8%)	137 (2.7%)
4	46 (0.5%)	23 (0.5%)	28 (0.5%)
5	16 (0.2%)	7 (0.2%)	12 (0.2%)
6	3 (< 0.1%)	1 (< 0.1%)	2 (< 0.1%)
7	4 (< 0.1%)	–	4 (< 0.1%)
10	1 (< 0.1%)	–	1 (< 0.1%)
Disease duration (years)	8.20 (8.31)	4.44 (6.69)	10.56 (8.34)

4.

## Abbreviations

MS: Multiple sclerosis
pwMS: People with multiple sclerosis
DMT: Disease-modifying therapy
mAb: Monoclonal antibodies
DMF: Dimethyl fumarate
RAT: Rapid-antigen testing
PCR: Polymerase chain reaction
EDSS: Expanded Disability Status Scale
